# Insights and Implications of Intricate Surface Charge Transfer and sp^3^-Defects in Graphene/Metal Oxide Interfaces

**DOI:** 10.1021/acsami.2c06626

**Published:** 2022-07-22

**Authors:** Daria Belotcerkovtceva, Renan P. Maciel, Elin Berggren, Ramu Maddu, Tapati Sarkar, Yaroslav O. Kvashnin, Danny Thonig, Andreas Lindblad, Olle Eriksson, M. Venkata Kamalakar

**Affiliations:** † Department of Physics and Astronomy, Uppsala University, P.O. Box 516, SE-751 20 Uppsala, Sweden; ‡ Department of Materials Science and Engineering, Uppsala University, P.O. Box 35, SE-751 03 Uppsala, Sweden; § School of Science and Technology, Örebro University, Fakultetsgatan 1, SE-70182 Örebro, Sweden

**Keywords:** graphene, charge transfer, graphene electronics, spintronics, sp^3^-defects

## Abstract

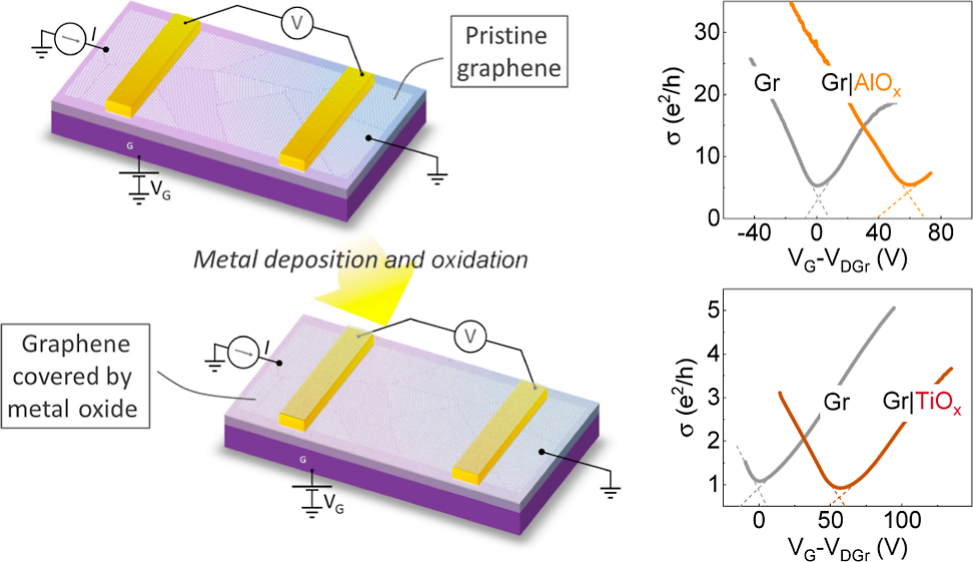

Adherence of metal oxides to graphene is of fundamental significance to graphene nanoelectronic and spintronic interfaces. Titanium oxide and aluminum oxide are two widely used tunnel barriers in such devices, which offer optimum interface resistance and distinct interface conditions that govern transport parameters and device performance. Here, we reveal a fundamental difference in how these metal oxides interface with graphene through electrical transport measurements and Raman and photoelectron spectroscopies, combined with ab initio electronic structure calculations of such interfaces. While both oxide layers cause surface charge transfer induced p-type doping in graphene, in sharp contrast to TiO_*x*_, the AlO_*x*_/graphene interface shows the presence of appreciable sp^3^ defects. Electronic structure calculations disclose that significant p-type doping occurs due to a combination of sp^3^ bonds formed between C and O atoms at the interface and possible slightly off-stoichiometric defects of the aluminum oxide layer. Furthermore, the sp^3^ hybridization at the AlO_*x*_/graphene interface leads to distinct magnetic moments of unsaturated bonds, which not only explicates the widely observed low spin-lifetimes in AlO_*x*_ barrier graphene spintronic devices but also suggests possibilities for new hybrid resistive switching and spin valves.

## Introduction

Graphene has evolved into an ideal medium for quantum and spin transport applications and a unique integration platform for complex heterostructures with other two-dimensional (2D) materials.^1,2^ Recent developments show record quantum and spin transport performance that can be achieved in large-scale chemical vapor deposited (CVD) graphene, making it a prospective material for practical implementation into quantum and spin-integrated circuits.^3−5^ Metal oxide interfaces are integral to graphene nanoelectronics and spintronic devices, ranging from memristors and single-electron transistors to tunnel field-effect transistors and graphene spin valves, where the interface nature guides their performance. In particular, titanium oxide and aluminum oxide have been widely used as tunnel barriers, primarily due to their efficacy and compatibility with device processing methods. Although ultrathin metal oxide layers can be directly realized using atomic-layer deposition or sputtering techniques, these techniques can lead to significant defects, impacting the overall structure and electrical nature of graphene.^6,7^ Instead, Ti and Al are deposited using electron beam evaporation in graphene spin devices and are subsequently oxidized upon contact with air/O_2_. In graphene electronic devices, metal oxides are used as gate dielectrics, effective barriers for graphene tunnel junctions,^8^ and thin-film memristive devices^9^ for practical imitation of synaptic activities, where interface charge transfer and bonding can guide the resistive switching phenomena.^10^ For both planar and vertical graphene spintronic devices,^11,12^ ultrathin layers (∼nm thickness) of the metal oxides serve as tunnel barriers offering optimum interface resistance to overcome the conductivity mismatch problem associated with electrical spin injection.^13,14^ However, obtaining faithful pinhole-free coverage is challenging for both aluminum and titanium oxides, prepared by e-beam metal evaporation and post-oxidation. In particular, compared to spin lifetimes up to ∼3.5 ns in graphene spintronic devices using TiO_*x*_ barriers,^15−17^ the AlO_*x*_-based devices widely show an order lower spin lifetime of ∼100 ps,^18−20^ which has been generally attributed to the presence of pinholes, consequent current crowding, and contact-induced spin relaxation.^21,22^ The one order lower performance of AlO_*x*_-based devices remains puzzling, despite the same fabrication process and similar thicknesses of oxides. Therefore, understanding the physics governing graphene/metal-oxide interfaces is of fundamental significance to graphene nanoelectronic and spintronic devices. While metals and metal oxides on graphene introduce surface charge transfer p-type or n-type doping arising from the difference in the work function of the metal relative to graphene,^17,23,24^ the situation is non-trivial for tunnel barrier oxides such as ultrathin TiO_*x*_ or AlO_*x*_ on graphene. Considering expected charge transfer effects, the redistribution of electron density after oxidation of metals is unclear, even from a theoretical perspective. In addition to surface charge-transfer doping, in-plane and out-of-plane defect-related doping in graphene can greatly influence charge and spin relaxation. The possibilities of introducing long-range and short-range scatterers, defects due to ultrathin oxide layers on graphene, and their direct influence on electrical properties of graphene and spin relaxation in tunnel transport through such barriers have never been explicit. This investigation aims to uncover, at an atomic level, the important differences in the nature of defects between Al oxide and Ti oxide adhering to graphene for their influence on electrical and spintronic performance.

## Materials and Methods

In this work, we explore the modifications in graphene due to its full coverage with metal oxides through electrical transport measurements, spectroscopic techniques, atomic force microscopy, and theoretical electronic structure calculations. To faithfully explore the extrinsic doping, defect effects, and their implications, we investigated the impact of TiO_*x*_ and AlO_*x*_ adsorption on the electronic properties of fully covered graphene. To understand the electronic alterations due to oxides, we measured electrical properties on the same devices before and after the oxide realization by metal deposition and oxidation. X-ray photoelectron spectroscopy (XPS) and Raman spectroscopy were used to determine the nature of doping and oxidation states of C, Al, and Ti in AlO_*x*_- and TiO_*x*_-covered graphene and their distinct interface behavior. The topography, probed by atomic force microscopy (AFM), shows characteristic features and coverage unique to each kind of oxide. We correlate these results with electronic structure calculations to understand the intricate interfacial properties and possible atomic and electronic configurations that lead to our experimental observations. Finally, we discuss how the interface defects observed in this study illuminate the current understanding of spin relaxation in the widely used tunnel barriers in graphene spintronics and lead to new implications for nanoelectronic and spintronic devices.

**Figure 1:**
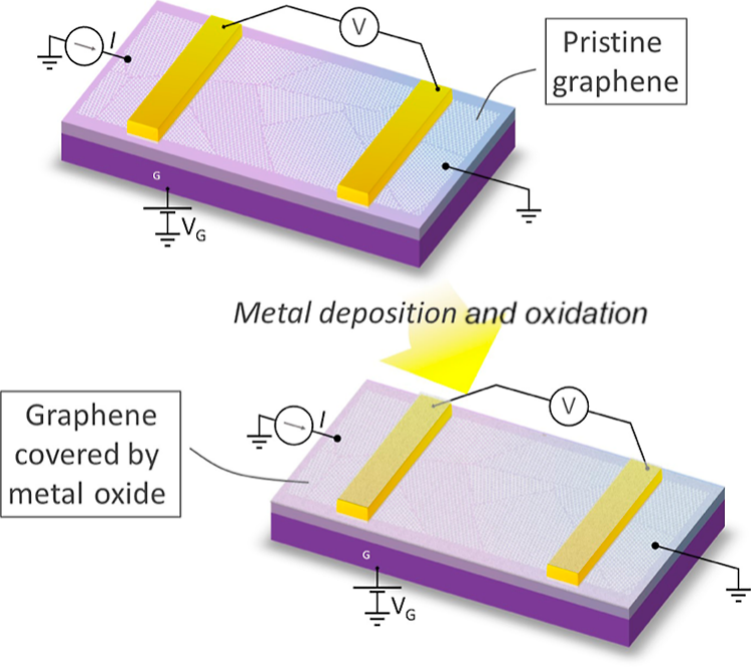
Experimental scheme of a graphene field-effect device before and after Al/Ti deposition and oxidation.

Figure 1 shows graphene field-effect devices used in our experiments before and after metal (metal oxide) realization on graphene. The first step in fabricating graphene devices is to pattern a graphene channel using optical lithography and Ar plasma etching, resulting in 5 μm wide graphene stripes. Following this, graphene devices were fabricated by electron beam lithography patterning, e-beam metal evaporation, and subsequent lift-off (details in the Supporting Information). Next, electrical measurements were performed on the resulting devices to obtain transport parameters of the pristine graphene channels. After these initial measurements, the devices were subjected to an additional layer of metal oxide by electron beam evaporation and oxidation in open air. The critical part of the procedure is the Ti or Al (0.8 nm) deposition on the same device and keeping the device working for repeated measurements with the metal oxide layers on top of graphene. This allowed us to understand the modification due to doping and possible defect creation in the same graphene stripes.

## Results and Discussion

**Figure 2:**
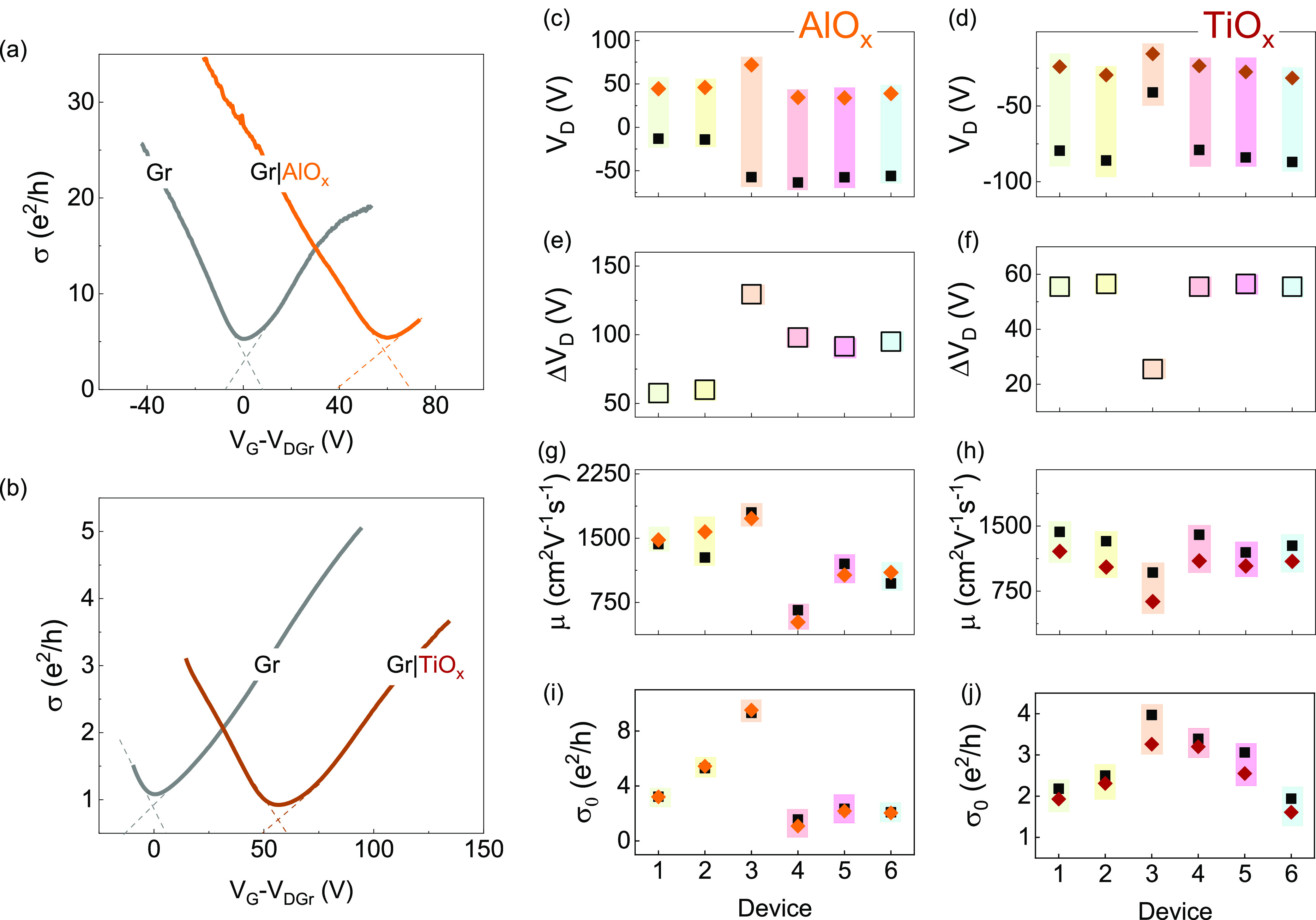
Electrical characteristics’ modification for graphene devices with AlO_*x*_ and TiO_*x*_ layers. Gate-dependent conductivity (in units of quantum of conductance *e*^2^/*h*) vs gate voltage (*V*_G_). Dirac curves for the devices with graphene before (gray curve) and after (orange/brown curve) deposition of (a) AlO_*x*_ and (b) TiO_*x*_. The dashed lines are provided here to guide Dirac point broadening. (c–j) Summary of Dirac point location (*V*_D_) and its shift (Δ*V*_D_), field-effect electron mobility (μ), minimum conductivity (σ_0_) for pristine graphene (dark square), and AlO_*x*_- and TiO_*x*_-deposited (colored diamond) devices.

The initial characterization of changes in the graphene properties was carried out by gate-dependent measurements of four-probe channel sheet-resistance (*R*_□_) with varying gate voltage (*V*_G_). To determine the contribution stemming from graphene and graphene covered with TiO_*x*_/AlO_*x*_, Dirac curves were measured on the same devices before and after the deposition of the metals. As shown in Figure 2a,b, for both oxide layers, the charge neutrality point (CNP), that is, the Dirac point (*V*_D_), shifts toward the positive gate voltage region. Such a shift means that these metal oxides cause a downward shift of the Fermi level in graphene, implying p-type doping. It is worth noting that the *V*_D_ shift (Δ*V*_D_) is significant even with an ultrathin ∼1 nm layer, and the interface trap density of states for the acceptor is affected by both types of oxides, as evidenced by Δ*V*_D_. In Figure 2c–j, all electrical parameters are compared with pristine graphene to determine changes in a total of 12 devices measured here. Despite the huge variation in *V*_D_ (i.e., doping level), minimum conductivity (σ_0_), and mobility (μ), both oxide-overlaid graphene samples show reasonably good values, and for most of the devices, the changes are moderately small (similar to or ∼10% for most devices) from initial pristine graphene devices to graphene/oxide devices. Strikingly, the sheet resistance at the CNP is similar for graphene before and after metal oxide realization, indicating a minimum conductivity that remains reasonably intact (as shown in Figure 2i,j). The minimum conductivity at *V*_D_ for both oxides (Figure 2i,j) showed less than 5% change from pristine graphene samples. Furthermore, in the shape of the Dirac curves, the plateaus around the CNP in the metal oxide-covered devices are not appreciably different from those seen in the pristine devices. The analysis of Dirac curves reveals (see Supporting Information Figure S1) a low trap density in all samples, and the minor changes in electrical parameters after the realization of top oxide layers can be linked to modifications in long-range Coulomb scattering due to charged impurities^25^ and the short-range defect scattering,^26^ including possible sp^3^ defects. Considering that Al and Ti metals result in n-type doping in graphene,^24,27,28^ these observations suggest that the p-type doping of ∼10^12^ to 10^13^ cm^–2^ (for change in Δ*V*_D_ of ∼ 50–100 V) is primarily due to the surface charge transfer between the oxide layers and graphene for most devices. This means that the oxide layer coverage achieved by electron beam evaporation and subsequent oxidation can be used to protect graphene without drastically altering its properties. The charge transfer serves as a tool to tune the carrier concentration faithfully. It is known that in-plane defects in graphene can lead to n-type doping,^29^ which rules out such defects. However, in addition to charge-transfer doping, sp^3^ out-of-plane defects could also lead to p-type doping^30^ and can cause spin-flip scattering as well as spin precession relaxation in graphene. To further explore the nature of such defects, we performed XPS measurements (see sample characterization details in the Supporting Information) on our samples.

**Figure 3:**
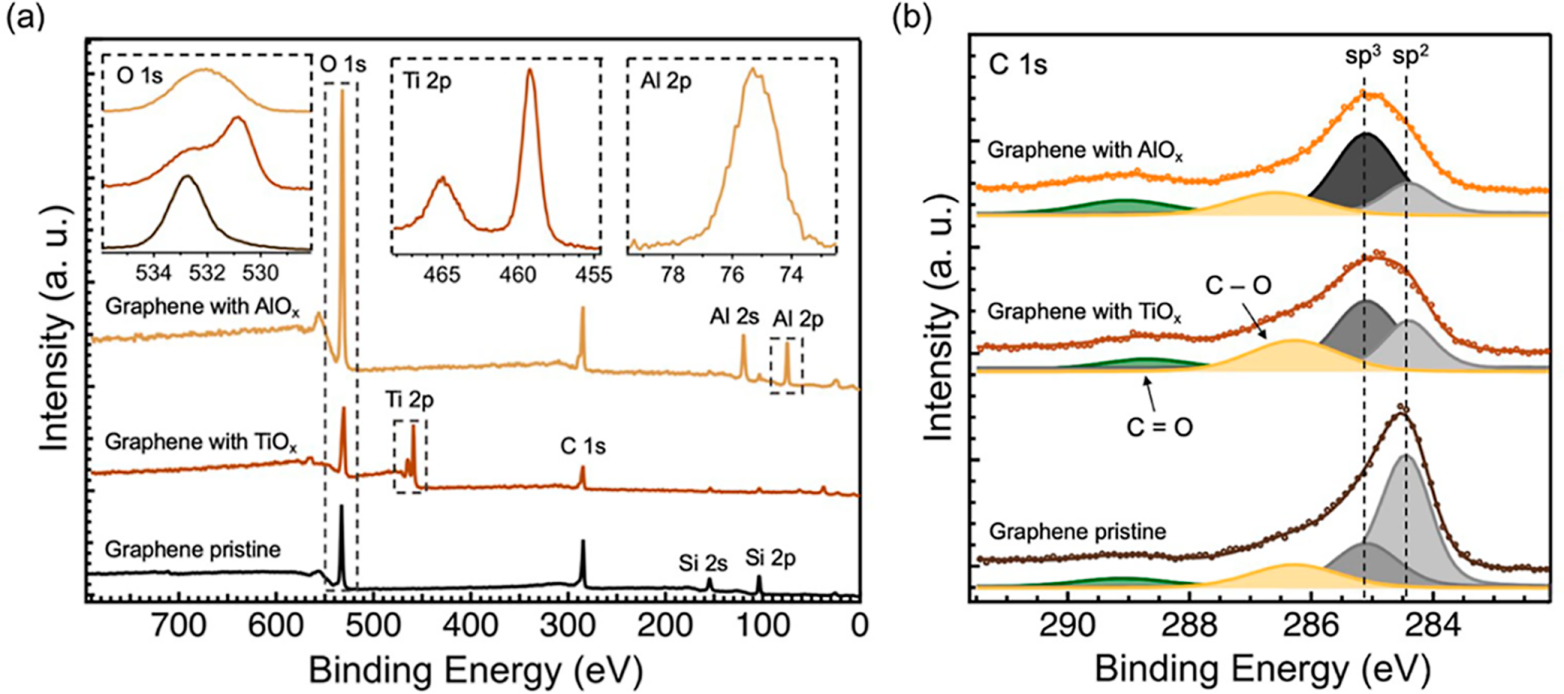
XPS characterization. (a) Overview spectra of pristine graphene (dark brown), graphene with deposited TiO_*x*_ (brown), and AlO_*x*_ (orange), and (b) C 1s components used for the least-squares fits. All spectra were obtained using a monochromatic Al K_α_ source. The binding energy is calibrated using the Si 2p peak at 103.3 eV in SiO_2_^37^ and the carbon sp^2^ peak at 284.4 eV.^38,39^

**Figure 4:**
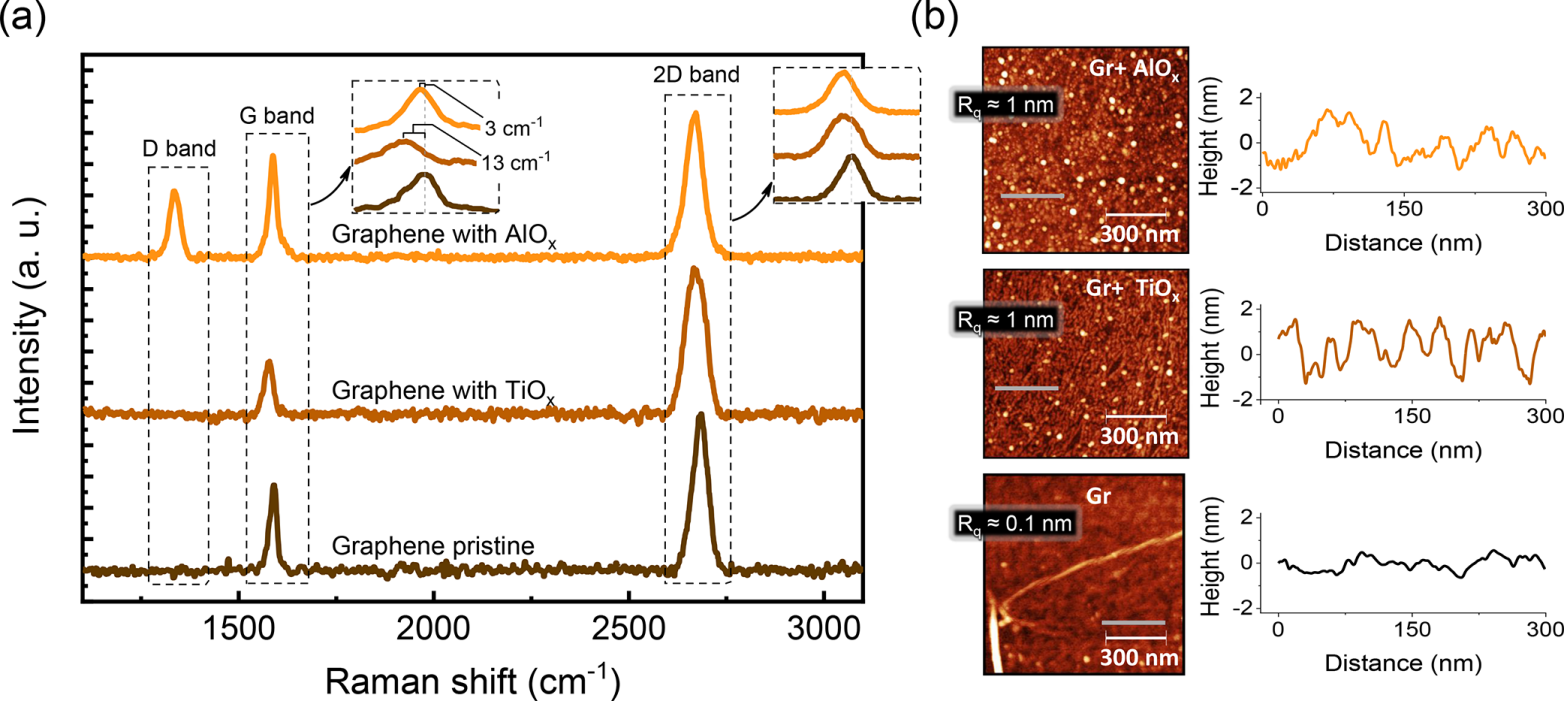
(a) Raman spectra of pristine graphene and graphene with deposited TiO_*x*_ and AlO_*x*_ along with insets showing shifts in G and 2D bands. (b) AFM images of representative graphene (Gr) and graphene after TiO_*x*_ (Gr + TiO_*x*_) and AlO_*x*_ (Gr + AlO_*x*_) deposition on top of it. The corresponding gray line scans show the roughness profiles, and *R*_q_ represents the average area roughness.

In Figure 3, the XPS data on pristine graphene and graphene with metal oxide layers are shown. The Al 2p spectrum (Figure 3a, upper right panel) shows a binding energy of 75 eV, suggesting that the deposited film is Al_2_O_3_.^31^ The regional spectrum of Ti 2p (Figure 3a, central panel) shows a binding energy of 459 eV for the Ti 2p_3/2_ core level, corresponding to TiO_2_.^32,33^ This implies that both metals are nearly fully oxidized. The XPS spectra for C 1s core-level binding energy is shown in Figure 3b. The observed increase in binding energy for oxide-covered samples suggests a charge transfer from graphene to interface atoms, confirming p-type doping. Figure 3b shows that the pristine graphene sample exhibits an expected intense sp^2^ contribution compared to graphene covered with metal oxides. The observed background contribution of sp^3^ can come from other sources, such as resist residue and other C-species in the sample measurement environment. In sharp contrast, the Gr-AlO_*x*_ sample shows a dominating contribution from sp^3^ defects (intensity ratio of sp^3^/sp^2^ ≈ 3), which signals strong evidence of sp^3^ defects created in the graphene lattice during the Al oxidation. Conversely, the transport parameters suggest that sp^2^ contribution should maintain dominance even for Gr-AlO_*x*_, so significant quenching of the signal of sp^2^ contribution can be due to the added surface oxide layers. Unlike Gr-AlO_*x*_ samples, we observe a lower value of sp^3^/sp^2^ ≈ 1.6 for Gr-TiO_*x*_, suggesting a lower-level source of the sp^3^ signal. Here, in our analysis, subtracting the sp^3^ contributions of bare graphene from our oxide samples can reveal some sp^3^ contribution also in Gr-metal oxide samples. However, since each sample has its own background, for comparisons, we chose to look at the sp^3^/sp^2^ intensity ratios. Considering that high spin lifetimes were achieved with TiO_*x*_ tunnel barriers, the increased sp^3^ intensity does not necessarily mean an sp^3^ hybridization of the graphene lattice.^34^ In fact, with a large 100 μm X-ray probe diameter, a significant sp^3^ contribution can originate from the PMMA resist residues and related effects intrinsic to the CVD graphene transfer process^35,36^ (similar to the sp^3^ background in pristine graphene). This, and the possible reduction in sp^2^ intensity due to oxide coverage, could increase the observed intensity at the sp^3^ binding energy for Gr-TiO_*x*_. Additionally, it is worth noting that surface carbon species bonded to oxygen atoms can have binding energies equivalent to those of sp^3^-carbon,^34^ which could further add to the surplus intensity at the sp^3^-binding energy position for the Gr-TiO_*x*_ sample. Thus, the XPS measurements with large-area sampling give qualitative evidence of the presence of sp^3^ carbon in the graphene lattice covered with AlO_*x*_. On the other hand, large-area sampling can be circumvented by micro-Raman characterization. Figure 4 shows the Raman spectra obtained on pristine graphene and graphene with metal oxide layers. First, as shown in Figure 4, for pristine graphene, the well-known G and 2D mode features, with frequencies near 1584 and 2678 cm^–1^, respectively, were obtained. The same peaks were also identified on the graphene samples with AlO_*x*_ or TiO_*x*_ layers, confirming the integrity of graphene sheets covered by oxide layers.

**Figure 5:**
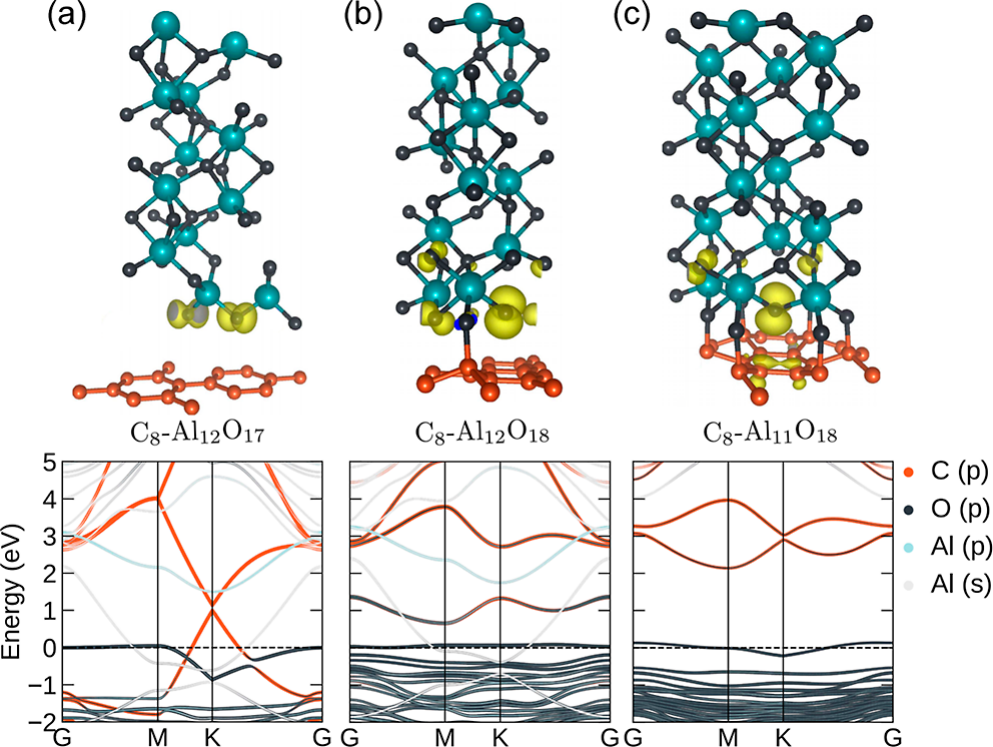
Simulated system’s geometry (upper panel). The atoms Al, O, and C are represented by green, gray, and orange, respectively. The yellow clouds are the atom-projected magnetic moment densities of the crystal. The lower panel shows the projected band structure of (a) slightly off-stoichiometric aluminum oxide on graphene monolayer structures (C_8_–Al_12_O_17_): the Fermi level shifts down in relation to the Dirac point and the unit cell magnetic moment is ∼0.44 μ_B_. (b) Perfect stoichiometry (C_8_–Al_12_O_18_) giving rise to partially sp^3^-bonded graphene leading to a local band gap opening and magnetic moments (0.61 μ_B_ per unit cell). (c) Electronic structure of graphene with sp^3^-bonded C_8_–Al_11_O_18_ where the unit cell magnetic moment is 1 μ_B_. The s- and p-bands from Al crossing both the Dirac cone and Fermi level (gray colored bands) are due to dangling bonds at the very top surface of the composite.

The ratio of the 2D peak to the G peak intensities (*I*_2D_/*I*_G_) is a parameter to determine the quality and monolayer structure of graphene.^40^ Here, in pristine graphene and graphene with TiO_*x*_, *I*_2D_/*I*_G_ ≥ 2 confirms the good quality of the CVD polycrystalline graphene that we employed in this study.^41^ However, in the presence of AlO_*x*_, graphene shows different behavior, with the value of *I*_2D_/*I*_G_ significantly decreasing to 1.45, which suggests possible degradation of the sp^2^ structure of graphene. In addition, the G peak’s doping sensitivity helps confirm the nature of doping. The location of the G peak for pristine graphene is 1590 cm^–1^. For graphene coated with AlO_*x*_, the peak shift was approximately 3 cm^–1^ and for the case of TiO_*x*_, it was approximately 13 cm^–1^ compared with pristine graphene. A similar shift was also observed for the 2D peak. Such a Raman shift to the left in the oxide-layered graphene indicates a p-type doping effect, which is in good agreement with our transport and XPS measurements. However, in the case of aluminum oxide-covered graphene, strikingly, we observed the emergence of the D peak (near 1340 cm^–1^), which is a signature of Raman-active defects in graphene, suggesting the introduction of appreciable sp^3^ carbon defects, a source of graphene modification by AlO_*x*_ only. Although Raman spectroscopy in most cases serves as a qualitative analysis, it is possible to quantify the presence of defects in graphene. Using the model proposed in literature,^42^ we obtained the concentration of defects for a graphene sample with AlO_*x*_ by extracting the G to D intensity ratio from the spectrum and the excitation laser wavelength (see Raman spectroscopy details in the Supporting Information). From the estimation, we found a concentration of defects for graphene with evaporated Al (0.8 nm) of ∼1.4 × 10^11^ cm^–2^, which is nearly one defect per 10,000 carbon atoms. As stated earlier, for a large shift in *V*_D_ of ∼ 50–100 V, a corresponding doping of 10^12^ to 10^13^ cm^–2^ can be expected. Therefore, the significant doping is due to surface charge-transfer doping, while the sp^3^-related defect contributions are up to 2 orders less, which is unique to the AlO_*x*_-interfaced graphene. Despite the defects, the electrical properties of graphene are relatively preserved in graphene/AlO_*x*_ devices. In graphene spintronic devices, the sp^3^ defects are expected to contribute to spin relaxation, mainly when the electrical spin injection is carried out in graphene spintronic devices, resulting in significantly shorter spin lifetimes. This has been conventionally attributed to pinholes in the AlO_*x*_ barrier. Density functional theory (DFT)-based electronic structure calculations showed that the interaction between graphene and metal oxides is weaker for Ti oxide than for Al oxide.^43^ The Ti-donated electron charge density is highly localized around the neighboring carbon atoms, and titanium does not tend to form clusters, which is excepted to lead to uniform coverage.^44^ To understand the surface morphology of the oxide coverage over graphene, we investigated the samples by AFM. We chose a 1 μm × 1 μm scan area to check the shape of the grains on top of the graphene surface and the height profile. Expectedly, there is a deviation from the smooth morphology of pristine graphene to graphene with the oxide layers. The common feature of oxide-covered graphene is the presence of ridges and grooves. The comparison between the surfaces is shown in Figure 4b. The AFM images show an alternation of dark and bright stripes in the case of Ti or circle-shaped dots in Al. The mean position was fixed to a 0 nm height in the height profile. Compared to the height profile with the standard topography of graphene, the oxide-covered graphene possesses a higher topographic root-mean-square roughness. While conspicuous pinholes can be identified from the deviation in the height profile and images for AlO_*x*_, perceptible swings in the height profile are also observed in the Gr-TiO_*x*_ system. One can attribute the other large clusters to possible resist residue regions observed in pristine graphene that can act as nucleation sites. Both oxides show an area roughness of *R*_q_ ∼ 1 nm, suggesting that both Ti and Al deposited on graphene by e-beam evaporation and following oxidation of the metals do not necessarily lead to full coverage, and hence, current crowding can be a common problem for both metal oxides. This leaves us with the additional sp^3^ defects unique to AlO_*x*_-layered graphene. Despite the XPS data indicating that Al and Ti are fully oxidized, we cannot assure perfect stoichiometric compositions leading to the observed charge-transfer doping in graphene. In particular, previous DFT calculations established charge transfer from graphene to titanium.^45,46^ However, the charge transfer and sp^3^ defect interface in graphene-Al oxide have not been addressed. To understand that on an atomic scale, we performed electronic structure calculations, shown in Figure 5.

Taking experimental conditions into account and using DFT calculations, we performed an interface study of the AlO_*x*_ corundum α-phase with a hexagonal unit cell in contact with a graphene monolayer sheet. The AlO_*x*_ slab structure can be arranged with different atomic terminations in proximity to graphene (e.g., oxygen or aluminum atom). Each case leads to varying results of the electronic property of the composite. Here, we only highlight three different interfaces (i.e., C_8_–Al_12_O_18_, C_8_–Al_11_O_18_, and C_8_–Al_12_O_17_) where oxygen is in proximity to graphene and the total energy suggests stable or metastable geometries. For all cases, the geometry was relaxed following force minimization. For other geometries and details, see the Supporting Information. Figure 5 shows atomic and electronic structure results for AlO_*x*_ layers on graphene using the experimental oxide layer thickness value of ∼1 nm. Notably, calculations performed for the perfect stoichiometry (C_8_–Al_12_O_18_), with graphene interfaced with aluminum atoms, did not yield any charge transfer and the Fermi level remained intact (see the Supporting Information). However, when oxygen atoms are in proximity to graphene, a strong hybridization between the p-orbitals from oxygen and p_*z*_-orbitals from carbon atoms causes the Dirac cone to be strongly distorted and positioned above the Fermi level.

Interestingly, calculations with a slight off-stoichiometry (Al_12_O_17_) give rise to systematic Fermi-level shifts of ∼1 eV and p-doping with oxygen atoms closer to the graphene lattice (Figure 5a) without completely distorting or destroying the Dirac cone. In the process of e-beam evaporation of Al, the possibility of Al atoms scattered from the graphene lattice and the activated carbon atoms being susceptible to subsequent bonding with oxygen atoms cannot be ruled out. Our calculations suggest that oxygen atoms can also make sp^3^ bonds with graphene, as shown in Figure 5b,c. The calculations reveal that p-type doping emerges due to the oxygen atoms next to the graphene layer. However, for stoichiometric and slightly off-stoichiometric Al_2_O_3_, even a few oxygen atoms firmly bound with the carbon atoms buckle the graphene layer locally and destroy the Dirac cone by inducing sp^3^ states and a local band gap opening. For computational reasons, the number of sp^3^ defects considered in the calculations is relatively high compared to experiments (where 1 out of 10,000 atoms have Raman-active sp^3^ defects). Nevertheless, the presented calculations show how sp^3^ bonds can induce significant band gap opening. The practical scenario for our samples prepared with CVD graphene can combine these two features with every 10,000^th^ location featuring a band structure as in Figure 5c. Overall, our experiments reveal that sp^3^-hybridized defect centers are a significant feature of AlO_*x*_, which is expected to buckle graphene locally. Such buckling is known to introduce spin–orbit coupling^47^ and magnetic moments.^48^ Our calculations with both perfect stoichiometric and non-stoichiometric Al-oxide sp^3^-bonded systems lead to an effective magnetic moment situated on unsaturated bonds, with values ranging from 0.44 μ_B_ (off-stoichiometric) to 1 μ_B_ (high-density sp^3^-bonded lattice) per unit cell.

In this investigation, we observed that non-invasive e-beam-evaporated Al and Ti-based oxides with metals near full-oxidized states adhere in subtle ways to graphene. While both oxides cause surface charge-transfer doping, the sp^3^ defects at the graphene/AlO_*x*_ interface can have significant implications for graphene spintronics, where spin currents are injected from a ferromagnet into graphene through the ultrathin oxide barrier. Spin–orbit coupling and local magnetic moments can dominate spin-flip and resonant scattering at graphene/AlO_*x*_ interfaces. Specifically, the resonant scattering with magnetic impurities has been considered responsible for observing low spin lifetimes of ∼100 ps, widely observed in AlO_*x*_ barrier graphene spintronic devices.^49^ With no magnetic ordering, the sparsely distributed sp^3^-based magnetic moments can become an additional source of spin relaxation in AlO_*x*_ barrier-based devices, other than the previously attributed pinholes in Al-oxide barrier-based studies in graphene spintronics. Furthermore, our calculations show that despite these magnetic moments being predominantly concentrated at non-saturated bonds (as shown in Figure 5), they can nevertheless induce magnetization in a small region of the graphene sheet, implying a significant source of spin scattering at the interface. In graphene spintronics, although hydrogenated graphene displayed increased spin lifetime with a normal g-factor,^50^ enhanced spin-scattering due to magnetic moment formation in graphene^51^ and reduced spin lifetimes due to colossal spin–orbit coupling^52^ have also been reported. Therefore, spin transport experiments with aluminum oxide-covered graphene could provide further insights into such observations. At the same time, the sparse magnetic moments at graphene/AlO_*x*_ interfaces could be ordered in exotic heterostructures via proximity effects^11^ for enhanced proximity-induced magnetism in graphene^11,53^ and in a controlled manner for novel spin valves. For resistive switching devices, the sp^3^ bonds can act as carrier traps for synaptic junctions, and oxygen ions in the AlO_*x*_ layer can be highly mobile in graphene and could form covalent bonds with the broken bonds of graphene for setting and resetting processes in memristive devices.^10^ Thus, magnetic defects with exchange-biased layers show potential for hybrid multilevel spin valve-resistive switching random access memory devices.

## Conclusions

In summary, we explored the subtle nature of ultrathin Al and Ti oxides interfacing with graphene. With similar coverage observed by AFM, electrical measurements revealed surface charge transfer p-type doping of ∼10^12^ to 10^13^ cm^–2^ for both metal oxides, with reasonably preserved charge mobility and sheet resistance. However, X-ray photoelectron spectroscopy suggests an intricate nature of the doping, not just due to charge transfer from C-atoms but also due to significant sp^3^ defects for Al-oxide. This is precisely confirmed by the emergence of the sp^3^ defect-active Raman D band for Al-oxide-layered graphene, in sharp contrast to Ti-oxide-layered graphene, where the band was absent. Our electronic structure calculations suggest that the observed 100 ppm sp^3^ defects originate from O bonding with C and an out-of-plane buckling of carbon atoms. Such defects in Al-oxide-layered graphene can lead to a local magnetic moment in ∼0.5–1.0 μ_B_, primarily located on unsaturated bonds of O atoms. In the Ti-oxide case, this moment is absent, which offers an explanation for the dramatic difference in spin lifetimes widely observed in devices with different oxide tunnel contacts. At the same time, these results provide new implications for developing unique interfaces for hybrid graphene resistive switching and spintronic devices.

## Supporting Information Available

Details of the device fabrication process; sample characterization using electrical measurements and analysis, Raman spectroscopy, AFM, and X-ray photoelectron spectroscopy; and computation details for aluminum oxide simulation with aluminum oxide interface construction, perfect stoichiometric AlO_*x*_/graphene interface, a small concentration of sp^3^ bond in perfect stoichiometric oxide, and slightly off-stoichiometric AlO_*x*_/graphene interface (PDF)
