# Supplementary material for: Insights and Implications of Intricate Surface Charge Transfer and sp3-Defects in Graphene/Metal Oxide Interfaces
Source: ACS Appl Mater Interfaces. 2022 Jul 22;14(31):36209–16. doi: 10.1021/acsami.2c06626 (PMC9376919; doi:10.1021/acsami.2c06626)
Supplement: Supplementary file 1 — am2c06626_si_001.pdf [file am2c06626_si_001.pdf]

Supporting information

Insights and implications of intricate surface charge transfer and sp^3-defects in graphene/metal oxide interfaces

Daria Belotcerkovtceva[1], Renan P. Maciel[1], Elin Berggren[1], Ramu Maddu[2], Tapati Sarkar[2], Yaroslav O. Kvashnin[1], Danny Thonig[1,3], Andreas Lindblad[1], Olle Eriksson[1,3], M. Venkata Kamalakar[1*]

[1]*Department of Physics and Astronomy, Uppsala University, Box 516, SE-751 20 Uppsala, Sweden*

[2]*Department of Materials Science and Engineering, Uppsala University, Box 35, SE-751 03 Uppsala, Sweden*

[3]*School of Science and Technology, Örebro University, Fakultetsgatan 1, SE-70182 Örebro, Sweden*

*Email: venkata.mutta@physics.uu.se

1 Device Fabrication

Commercial CVD graphene (Graphenea Inc.) grown on Cu substrate and transferred over 4 inches Si/SiO_2 wafer was employed for device fabrication. Via photolithography and 50 W argon plasma etching process, graphene was first patterned into 55 µm long stripes of 5 µm width. Then, the remaining photoresist was removed with acetone at 70°C and rinsed with isopropanol (IPA). Electrical contacts with a layered structure of TiO_x(2 nm)/Co(60 nm)/Al(3 nm) and separated by a spacing of 10 µm were fabricated on graphene stripes by e-beam lithography patterning and metal lift-off. The lift-off was achieved in hot acetone, rinsed by IPA. The devices were imaged using an optical microscope, with the optical parameters adjusted to observe the contrast of graphene over SiO_2/Si. After performing electrical measurements on pristine devices, the Ti or Al (0.8 nm) metal was evaporated by e-beam evaporation with a deposition rate of 0.5 Å/s, which was subsequently oxidized in air conditions.

2 Sample characterization

2a. Electrical Measurements

Electrical transport measurements were performed in high-vacuum conditions (~10^{-7} mbar) with a room-temperature setup, using a Keithley current source and a nanovoltmeter. In addition, gate voltage was applied by a Keithley source meter. Using the equation $\sigma\,(n) = Ce\left|\frac{n}{n_{imp}}\right| + \sigma_{res}$, where $C = 5 \times 10^{15}$ $V^{-1}s^{-1}$, n is the carrier density, e is the electronic charge and σ_{res} is the residual conductivity, we estimated the charge impurity density[1] (Fig. S1a shows a representative fitting for a sample before and after the realization of AlO_x) with a variation Δn_{imp} ~5-20 × 10^{11} cm^{-2} for both oxides, which is up to an order higher than the sp^3 defect density that we found using Raman spectroscopy. In addition, we also found low trap density ~10^{11} cm^{-2} (trapped carriers $n_{trap} = \Delta V_{DP}\, C_g/2e$ in the charge trapping effect[2], with a change in Dirac point ΔV_{DP} in hysteresis of Dirac curves, gate capacitance C_g, and electronic charge e) for all samples, compared to actual shift in the Dirac point due to oxide layers.

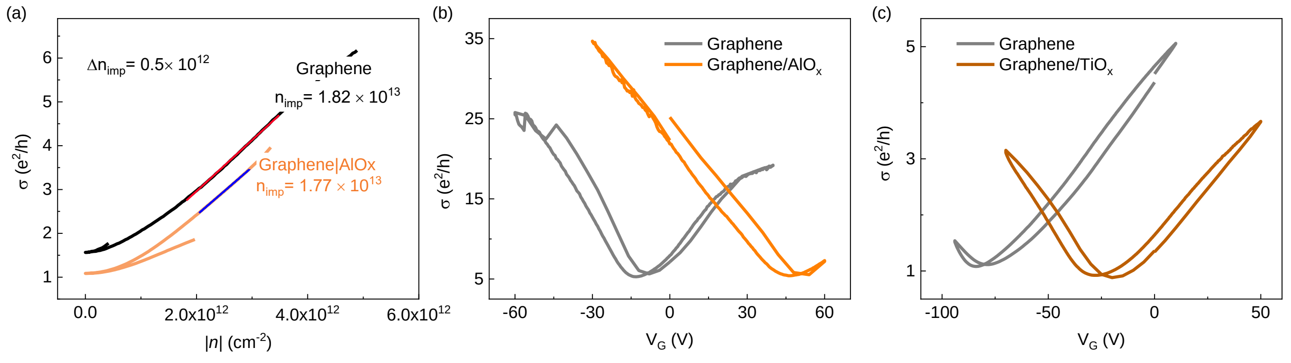

Figure S1. (a) Charge impurity density estimated by fitting the Dirac curves using $\sigma\,(n)$. Conductance change vs. gate voltage at 1 V/s gate sweep rate for graphene devices before and after oxide realization for (b) AlO_x and (c) TiO_x layers on graphene.

2b. Raman Spectroscopy

Raman spectroscopy was conducted with a Renishaw Reflex (Invia) Raman spectrometer for the single-layer CVD graphene (pristine graphene) and after Ti or Al deposition to see the evolution of the 2D and G peaks and the appearance of the D peak to calculate the defect concentration. To estimate the defect density via Raman spectroscopy, we used the empirical formula: ($n_D = \frac{2.4{*}10^{22}}{\lambda_L^4}\frac{I_D}{I_G}$ with λ_L the laser line wavelength) which is applicable in high-density regimes ($\frac{I_D}{I_G} \approx 60\%$)[3].

2c. Atomic Force Microscopy

Conventional AFM images of the graphene with deposited Ti and Al were obtained by Scanning Probe Microscope Bruker Dimension Icon with high resolution and low noise level for topography analysis using scan areas of 1 μm × 1 μm and 250 nm × 250 nm with corresponding height profiles and root mean square roughness. While imaging, we used PeakForce in the ScanAsyst imaging mode introduced by Bruker. The PeakForce method combines tapping mode as well as contact mode where direct force is controlled, and damaging lateral forces are avoided. The scan ScanAsyst algorithm optimizes the speed of the scan, z-position, and forces. In our samples, it was the optimum mode for high-resolution images of graphene with deposited ultra-thin metal oxides.

2d. X-ray Photoelectron Spectroscopy

X-ray photoelectron spectroscopy (XPS) was performed using a Physical Electronics Quantera II Scanning XPS Microprobe with a monochromatic Al Kα source (1486.7 eV). Overview spectra were collected with a beam diameter set to 200 μm and pass energy to 224 eV, resulting in an energy resolution of 1.3 eV. For single region spectra of O 1s, Al 2p, C 1s, and Ti 2p, a 100 μm beam diameter and 55 eV pass energy was used, resulting in an energy resolution of 0.7 eV. To avoid the possibility of defect contributions from the edges in the devices, we performed XPS measurements on a 0.7 × 0.7 cm^2 Si/SiO_2 substrate fully covered with CVD graphene and top metal-oxides. XPS spectra were analyzed by correcting the background with the Shirley method and fitting core-electron peaks with Voigt functions.

3 Computational details for aluminum oxide simulation

The ab-initio calculations were performed using the Vienna Ab-initio Simulation Package (VASP). For all simulations, we considered the projector augmented wave (PAW) approach and the generalized gradient approximation (GGA) using the Perdew-Burke-Ernzerhof (PBE) functional. With an energy cutoff of 520 eV, the plane-wave basis set for the AlO_x/graphene structure was chosen. The ionic forces were optimized with a Monkhorst-Pack mesh of k-points of 9 × 9 × 1 subdivisions, and the convergence criterion was 0.001 eV/Å. The vacuum space in the z-periodic direction was approximately 35 Å for all investigated configurations, which is large enough to avoid interactions between periodic images. The Van der Waals (vdW) interaction was considered for the combined graphene-oxide structures using the DFT-D3 approach with Becke-Johnson damping[4]. To analyze the electronic properties of the studied systems, we combined the vasp post-processing tools[5,6].

3a. Aluminum oxide interface construction

In this work, we considered a 2 × 2 × 1 hexagonal graphene supercell with $a_1 = a_2$ ~4.92 Å and a 1 × 1 × 1 aluminum oxide Al_2O_3 unit cell with lattice vectors $b_1 = b_2$ ~4.76 Å. To study the interface between graphene and aluminum oxide (α-Al_2O_3), we compensated for the existing lattice mismatch by applying a strain of 3.25% in the Al_2O_3 unit cell. Within this choice, we ensure that all graphene electrical-related properties are primarily preserved. In addition, an approximately 13 Å thick slab of Al_2O_3 (correspondent to the $Al_{12}O_{18}$ stoichiometry) is attached to the top of the graphene supercell in order to mimic the experimentally synthesized samples. For the simulation purpose, an initial spacing of 2 Å between these two crystals was considered.

The $Al_{12}O_{18}$ slab can have (two) Al or (three) oxygen atoms near graphene. Since one can find similar results even for different geometries, we consider the amount of dangling bonds at the top and at the bottom surface of the slab as a criterion to select the number of various interfaces to be studied. In particular, we chose three interfaces that are illustrated in Fig. S2a and S2c. Additionally, we investigated the structural and electrical properties of slightly off-stoichiometric oxides (Fig. S3) when one Al or O atom next to the graphene layer is removed.

3b. Perfect stoichiometric AlO_x/graphene interface

Fig. S2 shows the structure before and after the atomic relaxation, as well as the band structures of all considered graphene-$Al_{12}O_{18}$ systems, and summarizes the properties found for the combined structure graphene/$Al_{12}O_{18}$. In Fig. S2a, we observe that when aluminum is in proximity to the graphene layer, a flat bond between Al and O is formed, and the equilibrium distance between the two crystals exhibits an increase of 0.9 Å after force minimization. This suggests that this $Al_{12}O_{18}$ geometry has a weak interaction with graphene. Consequently, no significant change at the Dirac cone of the graphene structure is observed (see Fig. S2b). However, when oxygen is in proximity to the graphene layer, two (out of three) oxygen atoms form sp^3 bonds with the carbon atoms (Fig. S2c). The unbonded oxygen holds unpaired electrons. For this reason, this oxygen shows a total magnetic moment of 0.32 μ_B while the whole system has a magnetization of 0.61 μ_B per unit cell.

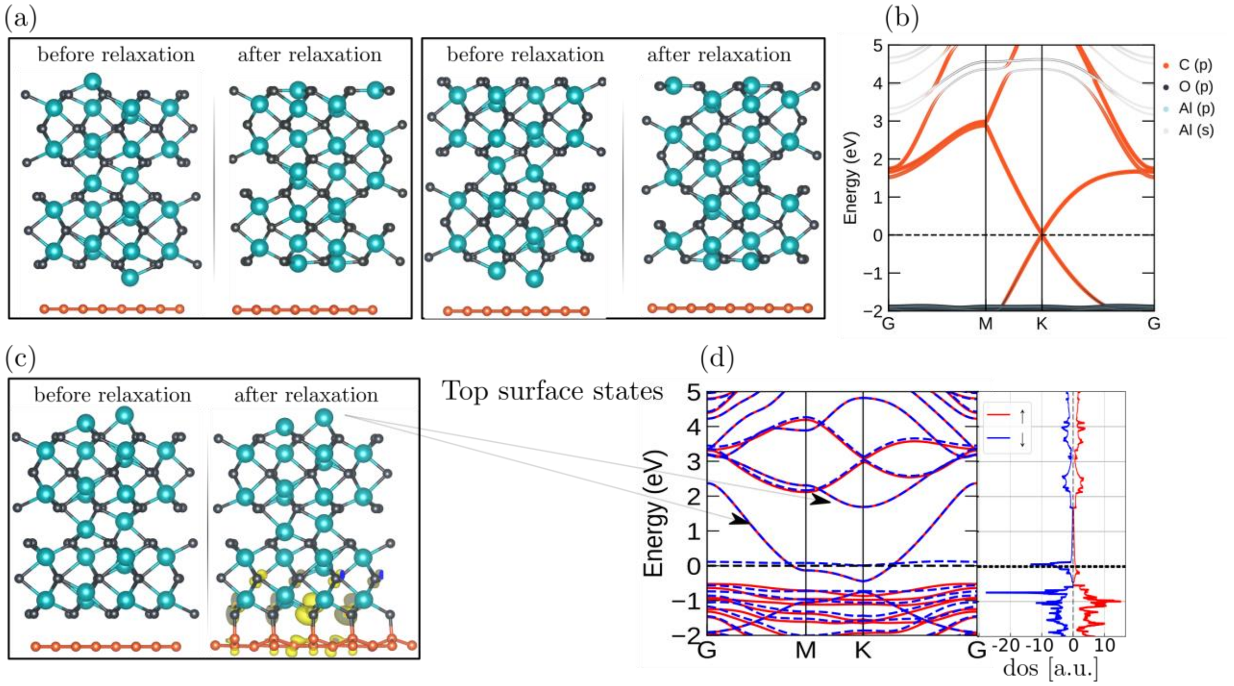

Figure S2. Scheme of the crystal structure interface (C_8-$Al_{12}O_{18}$) simulated for the perfect stoichiometric aluminum oxide. The colors green, gray, and orange represent Al, O, and C atoms. (a) Graphene/aluminum oxide interface before and after force minimization when aluminum is in proximity to the graphene layer. (b) The band structure shows no shifting of the Dirac cone when aluminum is in proximity to the graphene layer. (c) Force minimization when oxygen is in proximity to the graphene layer shows a formation of an sp^3 bond due to a strong hybridization between two oxygen and carbon atoms. The yellow crowds around O atoms represent the projected magnetic moment densities of the crystal. (d) The spin-polarized band structure and its corresponding total density of states (DOS) show the splitting of the different spins around the Fermi level. (The surface states' contributions from Al atoms are highlighted with arrows.)

3c. Small concentration of sp^3 bond in perfect stoichiometric oxide

To better understand the influence of the sp^3 bond in the electronic structure of pristine graphene, we created an interface with only one sp^3 bond per unit cell (rather than two, as shown in Fig. S2c). This interface is constructed by restricting the optimized system represented in Fig. S2c to just forming one sp^3 bond while preventing it from minimizing forces again. The resulting electronic band structure (see Fig. 5 in the main paper) has a significant gap widening of the Dirac cone. Furthermore, the magnetization of the entire system is lowered from 0.65 μ_B to 0.5 μ_B per unit cell.

3d. Slightly off-stoichiometric AlO_x/graphene interface

For the slightly off-stoichiometric system, we focused on the geometry where oxygen is in proximity to the graphene layer. The reason for such analysis is that the charge transfer is expected mainly from the carbon to the oxygen atoms. For the graphene-$Al_{11}O_{18}$ structure, we observe after force minimization that two sp^3 bonds are formed between the oxygen and carbon atoms (see Fig. S3a). However, in the case of graphene-$Al_{12}O_{17}$, the crystals exhibit an increase of 0.9 Å of the initial distance, and no deformation in the graphene layer is observed. The total magnetization corresponding to the graphene-$Al_{11}O_{18}$ structure is 1.0 μ_B, while for $Al_{12}O_{17}$ is 0.44 μ_B. A gap opening at the Dirac cone is observed for both systems (see Figs. S3b and S3d). Furthermore, in both the band structure and spin-polarized DOS, the magnetism around the oxygen atoms near to graphene layer causes the states around the Fermi level to have different spin polarities (see Fig. S3d). Despite this, we found that $Al_{12}O_{17}$ induces p-type doping in the graphene structure, whereas it opens a gap of approximately 1 eV.

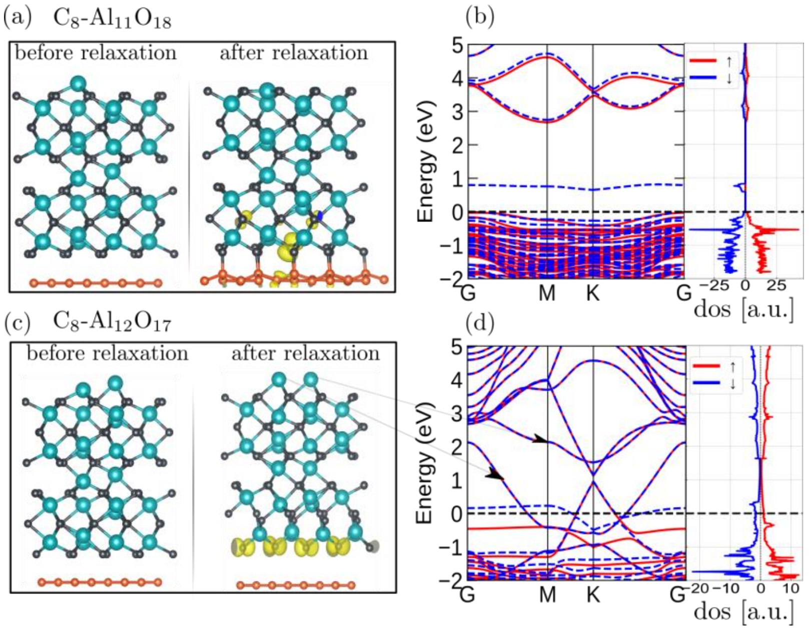

Figure S3. **Schematic representation of the slightly off-stoichiometric oxide.** The (a) $Al_{11}O_{18}$ and (c) $Al_{12}O_{17}$ slab before and after force minimization. The optimized structure also shows the projected magnetic moment densities of the crystal, primarily around oxygen atoms. The resolved band structure and the spin projected density of states of the (b) $Al_{11}O_{18}$ and (d) $Al_{12}O_{17}$, where red (blue) represents spin up (down). The arrows highlight the top surface states' contributions to the electronic band structure for the latter. (The representative colors of each atom, the yellow crowds around the oxygen atoms, and the arrows have the same meanings as in Fig. S2).

References

(1) Chen, J. H.; Jang, C.; Adam, S.; Fuhrer, M. S.; Williams, E. D.; Ishigami, M. Charged-Impurity Scattering in Graphene. *Nat. Phys.* **2008**, *4* (5), 377–381. https://doi.org/10.1038/nphys935.

(2) Kalon, G.; Jun Shin, Y.; Giang Truong, V.; Kalitsov, A.; Yang, H. The Role of Charge Traps in Inducing Hysteresis: Capacitance-Voltage Measurements on Top Gated Bilayer Graphene. *Appl. Phys. Lett.* **2011**, *99* (8), 1–4. https://doi.org/10.1063/1.3626854.

(3) Caņado, L. G.; Takai, K.; Enoki, T.; Endo, M.; Kim, Y. A.; Mizusaki, H.; Jorio, A.; Coelho, L. N.; Magalhães-Paniago, R.; Pimenta, M. A. General Equation for the Determination of the Crystallite Size La of Nanographite by Raman Spectroscopy. *Appl. Phys. Lett.* **2006**, *88* (16), 1–4. https://doi.org/10.1063/1.2196057.

(4) GRIMME, STEFAN, EHRLICH, STEPHAN, GOERIGK, L. Effect of the Damping Function in Dispersion Corrected Density Functional Theory STEFAN. *J. Comput. Chem.* **2011**, *32* (Sfb 858), 1456. https://doi.org/10.1002/jcc.

(5) M Ganose, A.; J Jackson, A.; O Scanlon, D. Sumo: Command-Line Tools for Plotting and Analysis of Periodic Ab Initio Calculations. *J. Open Source Softw.* **2018**, *3* (28), 717. https://doi.org/10.21105/joss.00717.

(6) Herath, U.; Tavadze, P.; He, X.; Bousquet, E.; Singh, S.; Muñoz, F.; Romero, A. H. PyProcar: A Python Library for Electronic Structure Pre/Post-Processing. *Comput. Phys. Commun.* **2020**, *251*. https://doi.org/10.1016/j.cpc.2019.107080.
